# The Liability of Hospital Committees under the Workmen's Compensation Act, 1906

**Published:** 1907-03-16

**Authors:** P. Michelli

**Affiliations:** Secretary. *Dreadnought* Hospital, Greenwich


### Editor's Letter-Box.

[Our Correspondents are reminded that prolixity is a great bar to publication, and that brevity of style and conciseness of statements greatly facilitate early insertion.]

### THE LIABILITY OF HOSPITAL COMMITTEES UNDER THE WORKMEN'S COMPENSATION ACT, 1906.

Sir,—The letter of Captain C. W. Dickinson, of the Royal Albert Hospital, Devonport, that appeared in your issue of March 2, should command the attention of hospital managers generally. There can be no doubt that some concerted action should be taken by the hospitals to protect their interests under the above Act. The conditions in charitable institutions vary considerably from those that exist elsewhere, and a policy covering all risks, not only to permanent, but to temporary officials, and to patients who may be engaged in assisting in the work of the hospitals, should be prepared so that we may have uniformity in this matter of insurance as in other matters.

I have been informed by more than one of the great insurance companies that if there was a combination among the hospitals to insure in one office of repute that office would distribute its liability, and that the insurance might be effected at a very much reduced rate.

There is the further question of the obligations of the insurance companies to the hospitals. The hospitals will render to the insurance companies a service which will save many thousands a year in claims—are not these services to be recognised in any substantial and systematic manner by the insurance companies? Besides, it seems but right and proper that any patient lying in hospital who ultimately receives compensation should be required to pay something towards the cost of his treatment, or that the insurance companies should be empowered to make an arrangement to this effect with the medical charities. If the injured man is doctored and nursed in his own home he will have to pay the doctor and nurse out of the insurance money—then why should he not do so when in hospital?

This subject was touched upon nearly a year ago in the *Hospital Gazette*, and has been mentioned in other periodicals, but so far the hospitals do not seem to have come to any concerted action. I had hoped that one of the great funds or the Central Hospital Council would have taken the matter up.

Yours faithfully,
P. Michelli, Secretary.

*Dreadnought* Hospital, Greenwich, March 8.

